# Increased Asymmetric Dimethylarginine in Severe Falciparum Malaria: Association with Impaired Nitric Oxide Bioavailability and Fatal Outcome

**DOI:** 10.1371/journal.ppat.1000868

**Published:** 2010-04-22

**Authors:** Tsin W. Yeo, Daniel A. Lampah, Emiliana Tjitra, Retno Gitawati, Christabelle J. Darcy, Catherine Jones, Enny Kenangalem, Yvette R. McNeil, Donald L. Granger, Bert K. Lopansri, J. Brice Weinberg, Ric N. Price, Stephen B. Duffull, David S. Celermajer, Nicholas M. Anstey

**Affiliations:** 1 International Health Division, Menzies School of Health Research and Charles Darwin University, Darwin, Northern Territory, Australia; 2 Menzies School of Health Research-National Institute of Health Research and Development Research Program, and District Ministry of Health, Timika, Papua, Indonesia; 3 National Institute of Health Research and Development, Jakarta, Indonesia; 4 Division of Infectious Diseases, University of Utah and VA Medical Centers, Salt Lake City, Utah, United States of America; 5 Division of Infectious Disease, Loyola University Medical Center, Maywood, Illinois, United States of America; 6 Division of Hematology-Oncology, Duke and VA Medical Centers, Durham, North Carolina, United States of America; 7 Centre for Vaccinology & Tropical Medicine, Nuffield Department of Clinical Medicine, John Radcliffe Hospital, Oxford, United Kingdom; 8 Division of Medicine, Royal Darwin Hospital, Darwin, Northern Territory, Australia; 9 School of Pharmacy, University of Otago, Dunedin, New Zealand; 10 Department of Medicine, University of Sydney and Department of Cardiology, Royal Prince Alfred Hospital, Sydney, New South Wales, Australia; Case Western Reserve University, United States of America

## Abstract

Asymmetrical dimethylarginine (ADMA), an endogenous inhibitor of nitric oxide synthase (NOS), is a predictor of mortality in critical illness. Severe malaria (SM) is associated with decreased NO bioavailability, but the contribution of ADMA to the pathogenesis of impaired NO bioavailability and adverse outcomes in malaria is unknown. In adults with and without falciparum malaria, we tested the hypotheses that plasma ADMA would be: 1) increased in proportion to disease severity, 2) associated with impaired vascular and pulmonary NO bioavailability and 3) independently associated with increased mortality. We assessed plasma dimethylarginines, exhaled NO concentrations and endothelial function in 49 patients with SM, 78 with moderately severe malaria (MSM) and 19 healthy controls (HC). Repeat ADMA and endothelial function measurements were performed in patients with SM. Multivariable regression was used to assess the effect of ADMA on mortality and NO bioavailability. Plasma ADMA was increased in SM patients (0.85 µM; 95% CI 0.74–0.96) compared to those with MSM (0.54 µM; 95%CI 0.5–0.56) and HCs (0.64 µM; 95%CI 0.58–0.70; p<0.001). ADMA was an independent predictor of mortality in SM patients with each micromolar elevation increasing the odds of death 18 fold (95% CI 2.0–181; p = 0.01). ADMA was independently associated with decreased exhaled NO (r_s_ = −0.31) and endothelial function (r_s_ = −0.32) in all malaria patients, and with reduced exhaled NO (r_s_ = −0.72) in those with SM. ADMA is increased in SM and associated with decreased vascular and pulmonary NO bioavailability. Inhibition of NOS by ADMA may contribute to increased mortality in severe malaria.

## Author Summary

Severe falciparum malaria is associated with impaired microvascular perfusion, lung injury and decreased bioavailability of nitric oxide (NO), but the causes of these processes are not fully understood. Asymmetrical dimethylarginine (ADMA), a competitive endogenous inhibitor of nitric oxide synthase (NOS), is an independent predictor of mortality in other critical illnesses, and can impair vascular function in chronic disease. ADMA can be produced by both the host and malaria parasites. The major novel findings of this study in malaria are that ADMA is an independent predictor of death in falciparum malaria, and is associated with decreased availability of nitric oxide in at least two organ systems affected by malaria parasites, the lining of blood vessels and the lungs. This study contributes to knowledge of regulation and availability of pulmonary and endothelial NO in critical illness and identifies pathogenic processes which may contribute to death in severe malaria. Therapies which increase the availability of NO or which reduce ADMA levels may have potential for adjunctive therapy of severe malaria.

## Introduction

*Plasmodium falciparum* causes ∼1 million deaths annually [1], [2]. Despite rapid parasite clearance with the anti-parasitic drug artesunate, the mortality rate in severe malaria remains high [3], [4]. Endothelial activation, parasite sequestration, impaired microvascular perfusion and dysregulated inflammatory responses are all thought to contribute to severe and fatal malaria [5]–[9]. Increased understanding of these pathogenic mechanisms may identify targets for adjunctive therapies to further improve outcomes.

Severe malaria is associated with impaired nitric oxide (NO) bioavailability and blood mononuclear cell NO synthase (NOS) type 2 expression in both children [10], [11] and adults [6]. The concentrations of L-arginine, the substrate for NO production by all three NOS isoforms [12], are low in children and adults with severe malaria and likely contribute to the decreased NO production found in severe disease [6], [10], [13]. However, in adults with moderately severe malaria, L-arginine concentrations are at least as low as those seen with severe malaria, yet there is no impairment of vascular and pulmonary NO bioavailability as found in severe disease [6]. This suggests that factors other than substrate limitation contribute to impaired NO bioavailability in severe malaria.

Asymmetrical dimethylarginine (ADMA) is a non-specific endogenous NOS inhibitor which decreases vascular function in cardiovascular and renal disease [14], [15]. Protein-arginine-methyltransferases methylate arginine residues in proteins and ADMA is released when these proteins undergo degradation [14]. ADMA is primarily eliminated by the enzyme dimethylarginine-dimethylaminohydrolase-1 (DDAH-1) in the liver and kidney, with ∼20% being excreted in the urine [16]. In adult sepsis, elevated ADMA is independently associated with increased mortality, a likely consequence of non-specific inhibition of homeostatic NO production [17], [18]. Increased protein catabolism with hepatic and renal dysfunction in severe malaria has the potential to increase ADMA and impair NO production, but the importance of ADMA in the pathogenesis of malaria is currently unknown. Clarification of the role of ADMA in malaria is of particular importance given a recent genome-wide association study in children linking DDAH polymorphisms with risk of severe malaria [19], and the potential for the parasite as well as host to produce ADMA [20].

Acute lung injury is a common but little-studied complication of severe falciparum malaria associated with high mortality [21]. In sepsis and critical illness, acute lung injury and mortality are associated with decreased total and pulmonary NO [22], [23]. Pulmonary diffusion capacity and exhaled NO concentrations are both reduced in severe malaria [6], [24], however the causative factors have not been identified. The role of ADMA in impairing pulmonary NO bioavailability in severe malaria, or indeed any critical illness, is not known.

In a prospective longitudinal study of Indonesian adults with malaria, we evaluated the hypotheses that concentrations of methylated arginines are independently associated with a) disease severity, b) reduced exhaled NO and vascular NO bioavailability and c) increased mortality.

## Results

### Patients

We measured asymmetrical dimethylarginine (ADMA), symmetrical dimethylarginine (SDMA) and L-arginine in 49 patients with severe malaria, 78 with moderately severe malaria and 19 healthy controls. In the SM patients, 20 patients had only one criterion for severe disease (coma in 14, hyperbilirubinemia in 4, respiratory distress in 2) with the remainder having >1 criteria. In total, 34 of the patients with SM were treated with intravenous artesunate and the remaining 15 received intravenous quinine [6]. All of the 78 MSM patients were treated with quinine with the exception of one who received artesunate. Exhaled NO concentrations (FeNO) could not be measured in those with coma, but were possible in 48% (11/23) of non-comatose SM patients, 88% (69/78) of MSM patients and 100% (19/19) of HCs. RH-PAT index was measured in all patients with malaria as well as HC. There were eight deaths among the patients with SM, and none in the MSM patients. Repeat RH-PAT and venous blood measurements were only performed in one and four of the eight fatal cases respectively. Baseline characteristics of study participants are summarized in **Table 1**.

**Table 1:** Baseline Characteristics of Patients According to Clinical Status.

	Healthy controls	Moderately-severe malaria	Severe malaria
Number	19	78	49
Age; mean (range), y	26 (18–40)	28 (18–56)	29 (18–56)
Males, No. (%)	13 (68)	32 (67)	36 (74)
Weight; mean (range), kg	60 (50–85)	58 (43–77)	57 (45–70)
Ethnicity, No. (%) Papuan highlander*	17 (89)	59 (77)	27 (55)
Current smoker, No. (%)	9 (47)	31 (40)	22 (45)
Days of fever before presentation; median (IQR)†	Nil	2 (1–5)	4 (1–7)
Systolic blood pressure; mean (range), mmHg†	128 (112–138)	110 (80–134)	105 (60–154)
Pulse rate; mean (range), beats/minute†	67 (48–91)	81 (54–118)	98 (61–138)
Respiratory rate; mean (range), breaths/minute†	19 (18–23)	23 (16–32)	30 (16–60)
Temperature; mean (range), °C†	35.6 (35.1–36.8)	36.5 (34.8–40.2)	37.2 (34.8–40.3)

* p<0.01 calculated by χ2 test.

† p<0.01 calculated by ANOVA or two sided *t* test.

IQR, interquartile range.

### ADMA, SDMA, L-arginine/ADMA ratio, ADMA/SDMA ratio and clinical disease

**Figure 1:**
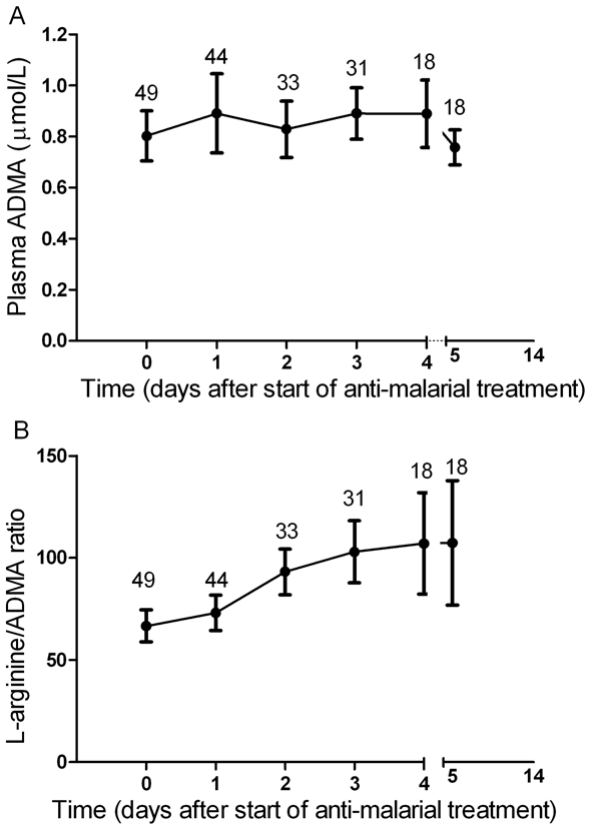
Plasma dimethylarginine concentrations in each group on enrollment (ANOVA: p<0.001). **A.** Plasma asymmetric dimethylarginine (ADMA) concentrations in each group on enrollment (ANOVA: p<0.001). In the severe malaria group, open circles represent fatal cases and closed circles survivors. Horizontal lines indicate mean for each group. **B.** Plasma symmetric dimethylarginine (SDMA) concentrations in each group on enrollment (ANOVA: p<0.001). In the severe malaria group, open circles represent fatal cases and closed circles survivors. Horizontal lines indicate mean for each group.

ADMA and SMDA concentrations were increased in SM patients (0.85 µM; 95% CI 0.74–0.96 and 1.67 µM; 95% CI 1.24–2.09 respectively) compared to those with MSM (0.54 µM; 95%CI 0.5–0.56 and 0.58 µM; 95%CI 0.54–0.63) and HC (0.64 µM; 95%CI 0.58–0.70 and 0.53 µM; 95%CI 0.47–0.59); ANOVA p<0.001 for both ADMA and SDMA, **Table 2**, **Figure 1A and 1B**. L-arginine concentrations were significantly higher in HCs compared to patients with MSM and SM, with the L-arginine/ADMA ratio decreasing with increasing disease severity (p<0.001; **Table 2**).

**Table 2:** Baseline Laboratory and Physiological Measurements According to Clinical Status.

	Healthy controls	Moderately-severe malaria	Severe malaria
Number	19	78	49
White blood cell count; mean (95% CI), x 10^3^µl^−1^ *	ND	5.9 (5.4–6.9)	9.5 (8.5–10.5)
Hemoglobin; mean (range), g/L*	ND	121 (70–170)	109 (60–163)
Plasma cell-free hemoglobin; median (IQR), µM†	1.3 (0.74–2.4)	2.6 (1.3–4.5)	5.4 (3.2–7.4)
Exhaled NO concentrations; median (IQR), ppb†	16.0 (9.5–19.3)	16.1 (10.7–25.3)	10.5 (7.5–15)
RH-PAT Index; mean (95%CI)*	1.87 (1.58–2.17)	1.82 (1.71–1.93)	1.37 (1.32–1.42)
Plasma creatinine; mean (95%CI), µmol/L*	ND	88 (82–94)	286 (207–365)
Plasma total bilirubin; mean (95%CI), µmol/L*	ND	16.7 (12.9–20.4)	95.2 (47.7–142.6)
Lactate concentration; mean (95%CI), mmol/L*	ND	1.4 (1.2–1.6)	2.93 (2.3–3.5)
Parasite density, geometric mean (range), µl^−1^ *	ND	14,900 (850–127,000)	35,100 (125–725,000)
HRP2 concentration; mean (range), log_e_ ng/ml*	ND	5.75 (1.34–8.79)	8.08 (1–10.98)
Soluble ICAM-1; mean (95%CI), pg/ml*	ND	569 (516–623)	938 (792–1084)
Soluble E-selectin; mean (95%CI), pg/ml*	ND	106 (95–118)	153 (113–193)
Plasma Angiopoietin-2; mean (95%CI), pg/ml*	2,800 (2,000–3,500)	6,500 (5,000–8,000)	17,000 (14,000–22,000)
Plasma ADMA; mean (95%CI), µmol/L*	0.64 (0.58–0.70)	0.55 (0.5–0.56)	0.85 (74–0.96)
Plasma SDMA; mean (95%CI), µmol/L*	0.53 (0.47–0.59)	0.58 (0.54–0.63)	1.67 (1.24–2.09)
Plasma L-arginine concentration; mean (95%CI) µmol/L*	77 (65–88)	41 (37–44)	49 (43–56)
L-arginine/ADMA ratio; mean (95%CI)*	121 (107–135)	78 (71–86)	64 (56–71)
Plasma Arginase; mean (95%CI), µmol/ml/h*	0.14 (0.08–0.17)	0.20 (0.14–0.24)	0.26 (0.22–0.31)

* p<0.01 calculated by ANOVA (overall) or two sided t test. ND, not measured.

† p<0.05 calculated by Kruskal-Wallis test.

IQR, Interquartile Range.

### ADMA, SDMA, L-arginine/ADMA ratio and exhaled nitric oxide concentrations

Exhaled nitric oxide concentration (FeNO) was significantly decreased in SM patients compared to MSM and HC; (**Table 2**). FeNO was inversely correlated with plasma ADMA concentration (r_s_ = −0.31, p = 0.003; **Table 3**) in all malaria patients, and in the subgroup of 11 patients with SM (r_s_ = −0.72, p = 0.01; **Table 3**). FeNO also correlated inversely with HRP2 concentration (r_s_ = −0.51, p<0.001) in malaria patients, but not in the SM group. FeNO remained inversely associated with ADMA, after adjusting for disease severity, creatinine and HRP2. FeNO was not associated with SDMA, the L-arginine/ADMA ratio, plasma hemoglobin or arginase.

**Table 3:** Correlation Coefficients (R_s_) for ADMA and Physiological Measures/Biomarkers of Severity.

		All malaria patients			Severe malaria		
		Correlation (r_s_)	p	df*	Correlation (r_s_)	p	df*
ADMA	RH-PAT Index	−0.32	0.001	139	−0.22	0.18	48
	Exhaled NO	−0.31	0.003	79	−0.72	0.01	10
	Lactate	0.30	0.01	124	0.1	0.3	48
	HRP2	0.40	<0.001	109	0.42	0.004	44
	ICAM-1	0.42	<0.001	109	0.61	<0.001	47
	E-selectin	0.12	0.4	116	0.20	0.10	47
	Ang-2	0.48	<0.001	140	0.56	<0.001	48
	TNF				0.29	0.06	39
	Creatinine	0.45	<0.001	117	0.55	<0.001	47
	Total Bilirubin	0.32	<0.001	115	0.58	<0.001	47

*df = degrees of freedom.

### ADMA, SDMA, L-arginine/ADMA ratio and endothelial function

As reported previously, the RH-PAT index was significantly lower in SM patients compared to those with MSM and controls (p<0.001) **Table 2**. In all malaria patients, there were moderate inverse associations between RH-PAT index and ADMA (r_s_ = −0.32; p<0.001; **Table 3**) and SDMA (r_s_ = −0.35; p<0.001) concentrations. After adjusting for factors previously shown to be associated with RH-PAT index including plasma hemoglobin [6], [7], [25], the inverse association with ADMA remained significant, with the final model including both ADMA and cell free hemoglobin (r = 0.40). In contrast, the L-arginine/ADMA ratio was not associated with the RH-PAT index. Longitudinally there was no association between the RH-PAT index and ADMA concentration or L-arginine/ADMA ratio in SM patients.

### ADMA, SDMA, L-arginine/ADMA ratio and markers of endothelial activation

Patients with SM had significantly elevated plasma concentrations of Ang-2, ICAM-1 and E-selectin compared to those with MSM and HC; **Table 2**. Angiopoietin-2 and ICAM-1 were significantly correlated with both ADMA (r_s_ = 0.48 and 0.42 respectively; p<0.001; **Table 3**), and SDMA (r_s_ = 0.54 and 0.52; p<0.001), and this was also apparent in the subgroup of SM patients; **Table 3**. ADMA remained independently associated with Ang-2 and ICAM-1 after adjusting for confounding factors, including creatinine, plasma hemoglobin, parasite biomass and disease severity.

There was no significant correlation between ADMA or SDMA with E-selectin, and none between the L-arginine/ADMA ratio and Ang-2, ICAM-1 and E-selectin.

### ADMA, SDMA, L-arginine/ADMA ratio and biomarkers of severity

The plasma creatinine, total bilirubin, *P. falciparum* histidine rich protein 2 (HRP2) and venous lactate were increased in SM compared to MSM (**Table 2**). In all patients with malaria, there were correlations between ADMA and SDMA with creatinine (r_s_ = 0.45; p<0.001; r_s_ = 0.69; p<0.001; **Table 3**), total bilirubin (r_s_ = 0.32; p<0.001; r_s_ = 0.36; p<0.001; **Table 3**), HRP2 (r_s_ = 0.46; p<0.001; r_s_ = 0.62; p<0.001; **Table 3**) and lactate (r_s_ = 0.3; p = 0.01; r_s_ = 0.29, p = 0.02; **Table 3**). The associations remained significant in SM patients for each of these biomarkers of disease severity except for venous lactate (**Table 3**). There was no association between L-arginine/ADMA ratio and any biomarker. TNF was only measured in the SM patients, and was associated with SDMA (r_s_ = 0.52; p = 0.001), but not with ADMA (p = 0.06).

### ADMA, SDMA, L-arginine/ADMA ratio and mortality

In SM patients, ADMA and SDMA concentrations were significantly higher in the 8 patients who died (1.28 µM; 95%CI 0.88–1.74 and 3.76 µM; 95%CI 1.88–5.56, respectively) compared to the 41 survivors (0.77 µM; 95%CI 0.64–0.84 and 1.27 µM; 95%CI 0.99–1.54, respectively; p<0.001; **Figure 1A and 1B**).

Each micromolar increase in ADMA and SDMA concentrations was associated with an 18-fold (OR 18.8 95% CI 2.0–181; p = 0.01) and three-fold (OR 3.0; 95% CI 1.5–6.2; p = 0.002) increased risk of death, respectively. ADMA but not SDMA remained a significant risk factor for death after adjusting for other confounding factors, such as Ang-2, creatinine, parasite biomass, bilirubin, base deficit and lactate. A final model predicting a fatal outcome included ADMA, Ang-2, HRP2 and creatinine (**Table 4**). The L-arginine/ADMA ratio was not associated with risk of death.

**Table 4:** Factors Associated with Death in Severe Malaria Patients.

Risk Factors	Univariate Model			Final Model		
	Odds Ratio	95%CI	P	Odds Ratio	95%CI	P
ADMA (µmol/L)	18.8	2.0–181	0.010	229	2.9–2675	0.025
Creatinine (µmol/L)	1.004	1.001–1.007	0.005	1.006	1.001–1.01	0.025
Angiopoietin 2 (pg/ml)	1.00058	1.00003–1.00109	0.024	1.00009	1.000002–1.0008	0.048
HRP2 (ng/ml)	1.00082	1.00009–1.001	0.01	1.0001	1.00002–1.002	0.017
Standard Base Deficit	1.19	1.01–1.4	0.037	0.93	0.64–1.35	0.659
Lactate (mmol/L)	1.32	1.02–1.41	0.044	1.29	0.51–3.3	0.485
Total Bilirubin (µmol/L)	1.006	1.001–1.009	0.015	1.003	0.98–1.1	0.21

**Figure 2:**
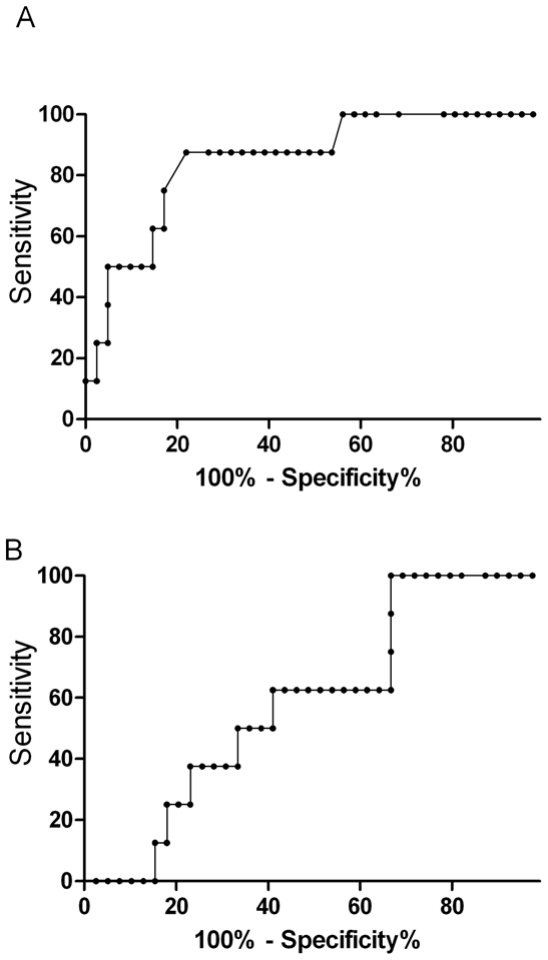
Top panel is the nonparametric receiver operating curve (ROC) assessing asymmetrical dimethylarginine (AUROC, 0.85; 95%CI 0.71–0.99) and the bottom panel venous lactate (AUROC 0.63; 95%CI 0.41–0.83) as prognostic markers for mortality in severe malaria (p = 0.003).

The prognostic value of ADMA in predicting a fatal outcome was measured by the area under the receiver operating curve (ROC). ADMA (AUROC 0.85; 95% CI 0.71–0.99; **Figure 2**) was comparable to other reliable prognostic indicators, including Ang-2 (AUROC 0.84; 95% CI 0.71–0.96), HRP2 (AUROC 0.86; 95% CI 0.73–0.94), base deficit (AUROC 0.73; 95% CI 0.53–0.92), and TNF (AUROC 0.71; 95% CI 0.43–0.98), and a better predictor of fatal outcome than venous blood lactate (AUROC 0.63; 95%CI 0.41–0.83; p = 0.003).

### Longitudinal Course of ADMA, SDMA and L-arginine/ADMA ratio

**Figure 3:**
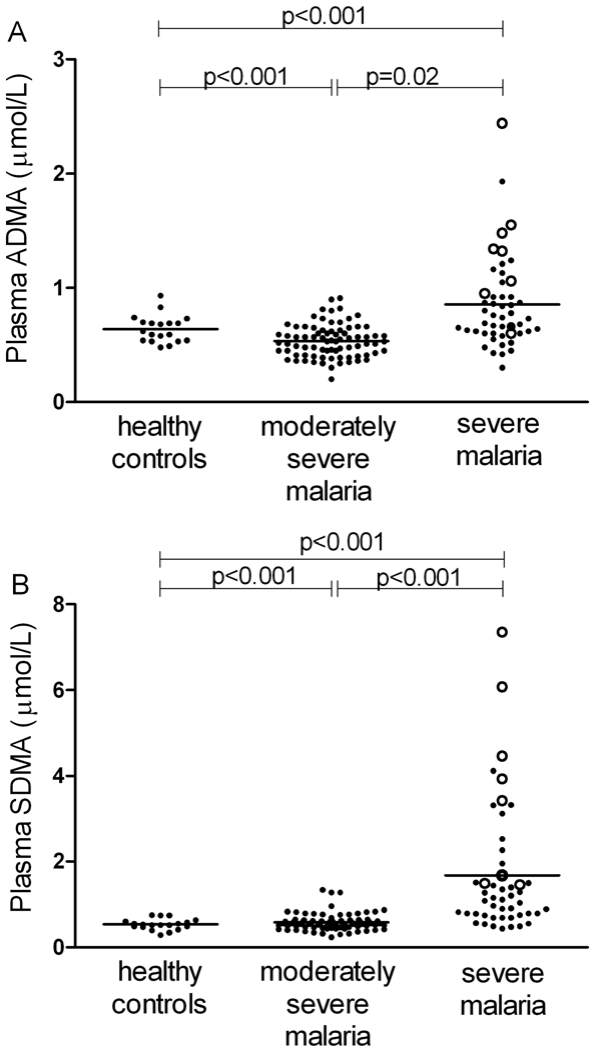
Longitudinal course of plasma ADMA concentrations and L-arginine/ADMA ratio in patients with severe malaria. Mean values (circles) and 95%CI (bars) are displayed at each time point. Values from day 5–14 indicate mean of all values obtained during this period. X axis values show time from start of anti-malarial therapy. Numbers indicate patients examined during each time period.

In patients with severe malaria, there was no significant change in ADMA (**Figure 3A**) or SDMA concentrations during the course of hospitalization among the overall group, survivors or those with a fatal outcome. Among survivors, there was a daily increase in L-arginine/ADMA (β = 9.1, p<0.001; **Figure 3B**) but no increase in those who died.

## Discussion

ADMA is increased in severe falciparum malaria and is an independent predictor of mortality. Indeed ADMA was a better predictor of death than blood lactate, previously shown to be a reliable prognostic indicator of increased mortality in severe malaria [26]. Our study demonstrated that elevated plasma ADMA concentrations are independently associated with decreased exhaled NO concentrations, impaired vascular NO bioavailability, increased endothelial activation and parasite biomass. To our knowledge this is the first demonstration of a relationship between increased ADMA and impaired exhaled NO in any critical illness. Taken together, these findings suggest that ADMA, an endogenous inhibitor of all three nitric oxide synthase (NOS) isoforms, reduces NO bioavailability in at least two organ systems and may contribute to increased mortality in falciparum malaria.

In critically ill patients, elevated ADMA concentrations are likely to result from increased production and reduced elimination. The elevation in both ADMA and SDMA may result from increased host production of methylated arginines in severe malaria. The majority of circulating ADMA is taken up by the liver before being metabolized by dimethylarginine-dimethylaminohydrolase-1 (DDAH-1); approximately 20% is excreted unchanged in the urine [16]. Hepatic blood flow is known to be significantly impaired in severe malaria [27]. The correlation of bilirubin and creatinine with increased ADMA, suggests that similar to sepsis [28], decreased hepatic and renal elimination may also increase ADMA concentrations in severe malaria. The large parasite biomass in severe malaria may also be a potential source of ADMA, with *Plasmodium falciparum* possessing protein arginine methyltransferases capable of producing ADMA [20]. The significant independent correlation between parasite biomass and ADMA on admission suggests this may be occurring *in vivo*, although the persistently elevated levels in severe malaria after commencement of anti-malarial therapy suggest the importance of altered host production and clearance in the post-treatment period. Increased clearance due to increases in either hepatic blood flow or DDAH activity may explain the decreased ADMA concentrations in patients with moderately severe malaria. There are no clinical studies to date documenting increased DDAH activity in mild inflammatory diseases, but hepatic blood flow is known to be significantly increased in acute uncomplicated falciparum malaria compared to patients with severe disease and healthy individuals [27]. The converse may also be true, with lower plasma ADMA concentrations potentially increasing vascular NO bioavailability in moderately severe malaria [14], and possibly contributing to elevated hepatic blood flow.

The loss of DDAH-1 function in a murine model of sepsis increased ADMA, reduced NO signaling, and worsened vascular pathophysiology including endothelial function [29]. In human severe sepsis, a polymorphism in the DDAH-2 enzyme increased ADMA levels which were associated with increased severity of organ failure and early septic shock [30]. Recently, a genome-wide association study in children found that a polymorphism in the gene encoding DDAH-1 was associated with an increased likelihood of severe malaria [19]. These studies indicate that altered DDAH function may be a contributor to organ damage and increased mortality in severe malaria as well as in other critical illnesses.

SDMA does not inhibit NOS, but competes with plasma L-arginine for intracellular uptake by the cationic amino acid transporters (CAT). Unlike ADMA, it is not metabolized by DDAH and is almost exclusively eliminated by the kidneys [16]. In chronic disease SDMA has recently been shown to be an independent predictor for major cardiovascular events in certain chronic diseases [31]. We find that in malaria, SDMA concentrations are associated with mortality and decreased vascular bioavailability on univariate analysis, but not after adjusting for renal function. While the association between SDMA and disease severity is likely to reflect the degree of renal impairment and SDMA retention, it is possible that retained SDMA may also contribute to decreased NO bioavailability in severe malaria.

In critically ill adults with organ failure and severe sepsis, ADMA concentrations are associated with increased all-cause mortality and the severity of organ failure [17], [30]. Investigators have hypothesized that this may result from non-selective inhibition by ADMA of all three isoforms of NOS, particularly homeostatic NOS3 (endothelial NOS) [18]. This is similar to the postulated mechanism to explain the increased mortality with use of N^G^-monomethyl-arginine (NMMA), another non-specific NOS inhibitor, in a phase 3 clinical trial of sepsis [32]. In falciparum malaria, systemic NO production is impaired in severe disease and hypoargininemia is likely to be a contributing cause [6], [10], [11], [13]. Exhaled and vascular NO are both reduced in adults with severe malaria [6], but not in moderately severe malaria (MSM) despite similar degrees of hypoargininemia [6]. This may be explained by the higher ADMA in severe malaria and a greater competitive inhibition of NOS in SM compared to MSM, similar to clinical studies of healthy volunteers in which ADMA infusion reduced blood flow [15], [33]. In mouse studies, ADMA infusion alone reduces splenic blood perfusion, but when combined with hypoargininemia, causes a reduction in renal, hepatic and splenic blood flow with organ damage [34]. Regulation of microcirculatory flow is dependent on pre-capillary arteriolar vasodilatory responses which in turn are critically dependent on NO production [35], with both likely to be decreased by ADMA in SM. By decreasing functional capillary density, ADMA could further impair microcirculatory function already compromised by parasite sequestration in capillaries and post-capillary venules [36].

We have previously shown that hemolysis-related NO quenching by cell-free hemoglobin is associated with reduced vascular NO bioavailability in severe malaria [25]. In malaria, increased ADMA and cell-free hemoglobin were independently related to endothelial dysfunction, suggesting that inhibition of NOS and NO quenching both reduce vascular NO bioavailability.

NO has multiple regulatory functions that maintain endothelial quiescence *in vitro*, including inhibition of endothelial Weibel-Palade body (WPB) exocytosis and ICAM-1 expression [37], [38]. Plasma concentrations of angiopoietin-2 (Ang-2), an angiogenic factor stored in WPBs, predict increased mortality in malaria [7], and ICAM-1 is a major endothelial adhesion receptor mediating cytoadherence of parasitized red cells and microvascular sequestration [5]. We demonstrate that ADMA levels correlate with increased Ang-2, but the association between ADMA and increased mortality is independent of Ang-2, suggesting effects of NO inhibition in addition to increased WPB exocytosis.

Acute lung injury is a complication of severe malaria in adults associated with a high mortality rate [21], [24]. Gas transfer at the alveolar-capillary membrane and exhaled NO are both decreased in severe falciparum malaria [6], [24]. In an animal model of sepsis–associated pulmonary injury, non-selective NOS inhibition causes increased lung edema [39]. Clinical studies have shown decreased pulmonary NO concentrations in patients with acute respiratory distress syndrome, as well as an association between decreased NO production and a worse outcome in acute lung injury [22], [23]. The lung is a major source of ADMA and increased concentrations are associated with pulmonary arterial hypertension [40], [41]. In severe malaria, both ADMA and parasite biomass are strongly inversely associated with exhaled NO concentrations, suggesting that both factors impair pulmonary NO production in severe disease.

There are several limitations in our study. Measurement of exhaled NO was not possible in patients with coma and was possible in only half of severe malaria patients without coma. Our results may therefore not reflect the relationship between ADMA and exhaled NO in all syndromes of severe malaria. In patients who died, only 4 of 8 had at least one repeat blood sample, and the longitudinal data may not truly reflect the course of the methylated arginines in fatal cases. RH-PAT index is at least 50% dependent on endothelial NO release [42], but we cannot exclude an effect of ADMA on other vasodilators such as prostacyclin and endothelium-derived hyperpolarizing factor. Although we have measured plasma ADMA concentrations, the effects of ADMA are intracellular. Nevertheless, *in vitro* studies with endothelial cells have shown that increasing extracellular ADMA results in five-fold increases in intracellular concentrations. This suggest that intracellular concentrations of ADMA in severe malaria may be higher, and may be adequate for meaningful inhibition of for all three NOS isoforms (IC_50_s ∼2-5µM) [43]. The observational nature of the study does not allow us to conclude with certainty a direct role for ADMA in the pathophysiology of severe malaria. While the association of ADMA with mortality may reflect impaired renal and hepatic function, it remained significant after adjusting for these factors in a multivariable model. Furthermore, increased ADMA from impaired hepatic and/or renal clearance is not just a marker of organ dysfunction in critical illness, with retained ADMA having functional consequences on NOS activity.

In summary, the endogenous non-selective NOS inhibitor ADMA is elevated in SM and is an independent risk factor for mortality. ADMA is also associated with decreased FeNO and vascular NO bioavailability, as well as increased endothelial activation and parasite biomass. Therapies which increase NO bioavailability or which diminish ADMA levels represent rational approaches for interventional trials of adjunctive therapy in severe malaria.

## Methods

### Study site and patients

The study was conducted at Mitra Masyarakat Hospital, Timika, Papua, Indonesia, a region with unstable transmission of multidrug resistant malaria [44], [45]. Written informed consent was obtained from all patients, if they were comatose or too ill, consent was obtained from relatives. The Ethics Committees of the National Institute of Health Research and Development, Indonesia, and Menzies School of Health Research, Australia approved the study.

Patients were ≥18 years old with moderately-severe (MSM) or severe (SM) *Plasmodium falciparum* malaria without *P. vivax* infection and with a hemoglobin level >60 g/L who had been prospectively enrolled in a study of endothelial dysfunction and exhaled NO [6]. Previous results from this study group have been published [6], [7], [13], [25]. Briefly, SM was defined as *P. falciparum* parasitemia and ≥1 modified WHO criterion of severity (excluding severe anemia). MSM was defined as fever within the preceding 48 hours, >1,000 asexual *P. falciparum* parasites/µL, no WHO warning signs or severe malaria criteria and a requirement for inpatient parenteral therapy because of inability to tolerate oral treatment. Healthy controls (HC) were non-related hospital visitors with no history of fever in last 48 hours, intercurrent illness or smoking in last 12 hours, or evidence of parasitemia [6]. Standardized history and physical examination were documented. Heparinized blood was collected daily, centrifuged within 30 minutes of collection and plasma stored at −70°C. Parasite counts were determined by microscopy. Hemoglobin, biochemistry, acid-base parameters and lactate were measured with a bedside analyser (i-STAT Corp). Patients were treated with anti-malarials and antibiotics using standard national protocols as previously described [6].

### L-arginine, asymmetrical dimethylarginine and symmetrical dimethylarginine

Solid phase extraction (SPE) of amino acids was followed by derivatisation with Accq-Fluor and separation on a Gemini-NX column at pH 9 [46]. The SPE method gives absolute recoveries of >80% for ADMA and symmetrical dimethylarginine (SDMA) and average relative recoveries of 102% for ADMA and 101% for SDMA. The HPLC method gives intra-assay RSDs of 2.1% and 2.3% and inter-assay RSDs of 2.7% and 3.1% for ADMA and SDMA respectively [46].

### Cell-free hemoglobin, cytokines, endothelial activation, arginase and L-arginine

Plasma concentrations of cell-free hemoglobin and the endothelial activation markers, ICAM-1, E-selectin and angiopoietin-2 were measured by ELISA as previously reported in this population [6], [7], [25]. Total parasite biomass was quantified by measuring plasma histidine rich protein-2 (HRP2) by ELISA [6], plasma arginase by a radiometric method [6] and plasma TNF concentrations by flow cytometry, as previously reported [7].

### Endothelial function and pulmonary nitric oxide

Endothelial function was measured non-invasively using peripheral arterial tonometry (EndoPAT) by the change in digital pulse wave amplitude in response to reactive hyperemia, giving a RH-PAT Index as reported previously [6]. The RH-PAT index is at least 50% dependent on endothelial NO production [42]. Endothelial function was measured daily until death or discharge, or until the RH-PAT index was above an *a priori* cutoff (1.67) for two consecutive days [13]. Fractional concentrations of exhaled NO were measured by NO analyser (Aerocrine), as previously described, using American Thoracic Society guidelines and a flow rate of 250 ml/sec [6].

### Statistical methods

Statistical analysis was performed with STATA 9.2 software. Intergroup differences were compared by ANOVA or Kruskal-Wallis test, where appropriate. Pearson's or Spearman's correlation coefficients were determined depending on normality of distributions. Multiple stepwise linear regression was used to adjust for confounding variables. Longitudinal associations were assessed by mixed effects modeling using generalized estimating equations. Logistic regression was used to determine the association between death and ADMA concentrations. Variables hypothesized, as well as those shown in previous publications [6], [7], [25], to contribute to mortality, pulmonary NO and endothelial pathology were included in a multiple regression model if p<0.05 on univariate analysis and retained if they remained significant. Goodness-of-fit was assessed by the Hosmer-Lemeshow goodness of fit test and independent variables tested for interactions. The prognostic utility of continuous variables was measured using the area under the receiver operating curves (ROCs) and its 95% confidence intervals were calculated. A two-sided value of p<0.05 was considered significant.
