# Correction: Increased Asymmetric Dimethylarginine in Severe Falciparum Malaria: Association with Impaired Nitric Oxide Bioavailability and Fatal Outcome

**DOI:** 10.1371/annotation/e49842d3-ea72-45a4-93d4-2f494baee962

**Published:** 2010-05-11

**Authors:** Tsin W. Yeo, Daniel A. Lampah, Emiliana Tjitra, Retno Gitawati, Christabelle J. Darcy, Catherine Jones, Enny Kenangalem, Yvette R. McNeil, Donald L. Granger, Bert K. Lopansri, J. Brice Weinberg, Ric N. Price, Stephen B. Duffull, David S. Celermajer, Nicholas M. Anstey

The images for Figures 1 and 3 were incorrectly switched. The image that appears as Figure 1 should be Figure 3, and the image that appears as Figure 3 should be Figure 1. The figure legends appear in the correct order.

